# “We have nice policies but…”: implementation gaps in the Ghana adolescent health service policy and strategy (2016–2020)

**DOI:** 10.3389/fpubh.2023.1198150

**Published:** 2023-12-12

**Authors:** Emelia Afi Agblevor, Natasha Afua Darko, Priscilla Ama Acquah, Selasie Addom, Tolib Mirzoev, Irene Akua Agyepong

**Affiliations:** ^1^Faculty of Public Health, Ghana College of Physicians and Surgeons, Accra, Ghana; ^2^Dodowa Health Research Centre, Dodowa, Ghana; ^3^London School of Hygiene and Tropical Medicine, London, United Kingdom

**Keywords:** adolescents, youth, sexual health, reproductive health, mental wellbeing, mental health, Ghana, implementation

## Abstract

**Introduction:**

Although policies for adolescent health exist in Ghana, their implementation is challenging. Availability of services for adolescent sexual and reproductive health and adolescent mental health remains less than desired, with adolescent mental health being particularly neglected despite being an important contributor to poor health outcomes. This study presents an analysis of gaps in the implementation of the Ghana Adolescent Health Service Policy and Strategy (2016–2020), including how and why the context influenced the observed implementation gaps.

**Methods:**

Data for this study is drawn from 17 in-depth interviews with purposefully identified key stakeholders in adolescent mental, sexual, and reproductive health across the national and subnational levels; four focus group discussions (FGDs) with district health management teams; and 11 FGDs with adolescents in and out of schools in four selected districts in the Greater Accra region. Data were analyzed using both inductive and deductive approaches. The deductive analysis drew on Leichter’s conceptualization of context as structural, cultural, situational, and environmental factors.

**Results:**

Of the 23 planned strategies and programs for implementing the policy, 13 (57%) were partially implemented, 6 (26%) were not implemented at all, and only 4 (17%) were fully implemented. Multiple contextual factors constrained the policy implementation and contributed to the majority of strategies not being implemented or partially implemented. These factors included a lack of financial resources for implementation at all levels of the health system and the related high dependence on external funding for policy implementation. Service delivery for adolescent mental health, and adolescent sexual and reproductive health, appeared to be disconnected from the delivery of other health services, which resulted in weak or low cohesion with other interventions within the health system.

**Discussion:**

Bottom-up approaches that engage closely with adolescent perspectives and consider structural and cultural contexts are essential for effective policy implementation. It is also important to apply systemic and multi-sectoral approaches that avoid fragmentation and synergistically integrate policy interventions.

## 1 Introduction

Globally, 1.3 billion adolescents comprise 16% of the world’s population (1). An estimated 23% of the population in West and Central Africa are adolescents, and this percentage is predicted to grow to 32% between 2019 and 2030 (2). In Ghana, where this study was conducted, adolescents (10–19 years) comprise approximately 22% of the population (3).

Adolescence, the period between childhood and adulthood (ages 10–19 years), is a very turbulent period marked by various physiological, psychological, physical, and social changes and sexual development. This is also the phase where habits are formed in relation to diet, physical activity, substance abuse, and sexual activity, which can safeguard the health of adolescents or put them at risk in the future (1). According to the World Health Organisation (WHO), 1.1 million adolescents die each year, with the leading causes of death being road injuries, suicide, and interpersonal violence (4). In terms of years lost to disability (YLD), the five top causes among 10–14-year-olds are unipolar depressive disorders, iron deficiency anemia, asthma, back and neck pain, and anxiety disorders. This picture is similar for 15–19-year-olds, with the only difference being that asthma is replaced by alcohol use disorders among men (5). Adolescent health should be a global health priority as it will have profound implications for social, political, and economic development.

While adolescent pregnancy, childbirth, and early child marriage have declined globally, these remain disproportionately high within the West African sub-region (6). According to the Ghana District Health Information Management System (DHIMS), in 2020, a total of 109,888 teenage pregnancies were recorded, with 2,865 being among girls aged 10–14 years. These may be underestimated since home deliveries, which are not captured in the DHIMS, still occur in Ghana. Similarly, child marriages continue to be high, with 79,733 girls aged 12–17 years reported to be married or already living with a man, thus contributing to high incidences of adolescent pregnancy and childbirth (7).

Adolescent health and wellbeing is a stated priority in West Africa. In 2016, the West Africa Health Organisation (WAHO), the health branch of the Economic Community of West African States (ECOWAS), developed an orientation manual for the development of national strategies for adolescent and youth health in the countries of ECOWAS (8). The manual aims to help governments develop or update national strategies, policies, and programs for adolescent and youth health, including Adolescent Sexual and Reproductive Health (ASRH) policies. In Ghana, the Adolescent Health Service Policy and Strategy (2016–2020) mirrors the strategies and policies for adolescent health in the ECOWAS manual with a focus on sexual and reproductive health, HIV/STIs, mental health conditions, communicable and non-communicable diseases, interpersonal violence, as well as intentional and unintentional injuries (9).

Mental health has been and remains a neglected priority in Ghana and globally. Despite the introduction of community-based mental health workers in 2007, access to mental health services remains generally low at the primary healthcare level, and access is even lower for adolescents (10, 11). However, mental health services for adolescents are very important, as approximately 75% of mental health conditions develop in the period of adolescence (12). Several studies have shown that unresolved mental health conditions experienced during adolescence continue into adulthood and impact other health outcomes (13, 14). Furthermore, poor mental health is often related to other health and developmental issues, such as low educational achievements, substance abuse, violence, and poor reproductive and sexual health (12). Thus, it is imperative that mental health services to adolescents are prioritized to mitigate the triggers that can lead to risky sexual behaviors, substance abuse, low educational achievements, violence, and poor nutrition (12).

The Adolescent Health Service Policy and Strategy (AHSPS) (2016–2020) was developed by the Ghana Health Service and the Ministry of Health, with inputs from the National Population Council, Ghana Education Service, National Youth Authority, the National Commission for People with Disabilities, and various non-governmental organizations and development partners. The vision of the AHSPS (2016–2020) was “*to improve the health status of adolescents and young people through equitable access to appropriate, comprehensive, gender-sensitive, quality and cost-effective adolescent and youth responsive health information, education and services*” (9).

Despite the existence of this comprehensive adolescent health policy in Ghana, observation suggests that many challenges the policy was aimed to address continue to persist, resulting in several gaps between the stated policy objectives and what occurs in practice. Understanding how the policy was implemented and why policy implementation gaps persist is necessary to inform future policies with lessons learned.

Several studies have explored different aspects of policies and interventions for adolescent health that may help explain gaps in policy implementation; however, most focused on only specific aspects of adolescent health. These included analyses of barriers and facilitators of implementation of policies for reducing adolescent pregnancy in Ghana (15), barriers to access and provision of adolescent sexual and reproductive health services (16–18), care-seeking behaviors and how that affects the provision of mental health (19), barriers to low uptake of contraception (20), and awareness of rights with regards to reproductive health (21–24). Thus, the literature contains a somewhat fragmented narrative that covers particular interventions or challenges rather than convey a comprehensive analysis of a holistic national policy to understand how and why the policy was implemented within its specific context. Context can nonetheless be an important part of the explanation of the “how and why” of policy implementation gaps and therefore requires careful analysis.

The objective of this study is to report the analysis of the Ghana Adolescent Health Service Policy and Strategy (2016–2020), including its contents, how the policy was implemented, which gaps in implementation were observed, and the important role of context in policy implementation. This study is drawn from a larger multi-country study on adolescent health in West Africa, which aimed to understand how and why adolescent mental, sexual, and reproductive health and wellbeing policy and program priorities are implemented at the primary care level in Ghana, Niger, and Burkina Faso. We hope that it will be of relevance to those interested and engaged specifically in improving adolescent health in similar settings and advancing understanding of policy implementation more generally.

## 2 Methods

### 2.1 Study design

The study was a qualitative single case study design. We defined the case as a national adolescent health policy with a completed implementation period. We purposefully chose the Ghana Adolescent Health Service and Policy Strategy document (2016–2020), after mapping different policy documents and legislation on adolescent health in the 10 years preceding the study (2011–2021). At the time of the study, the Adolescent Health Service and Policy Strategy document (2016–2020) met the eligibility criteria of a contemporary policy with a completed implementation policy period.

### 2.2 Study setting

The study was conducted in the Greater Accra region, one of 16 administrative regions of Ghana. Greater Accra is the most urbanized region in the country, with 91.7% of its total population living in urban centers (3). The Greater Accra was purposively selected because of three considerations. First, it comprises people with diverse cultural, religious, educational, and ethnic backgrounds, thus making it an ideal setting for the analysis of policy implementation. Second, Greater Accra is the administrative capital of Ghana, and it was easy to access relevant national and sub-national level stakeholders who were involved in the development and implementation of the AHSPS document. Third, it is the region where the research institution was based, thus making the study feasible within available resources.

Four districts were purposively selected within the region to aid the understanding of how the policy was implemented at the primary care level. Criteria for selection were driven by our intention to get a mix of the varying district contexts of highly urbanized, rural, and peri-urban, as well as districts where the Ghana Education Service and Ghana Health Service authorities allowed access for the research team to work in.

### 2.3 Methods and participants

The data was collected between June 2021 and December 2022 using a combination of multiple qualitative methods. These included desk review of policy documents, key informant in-depth interviews, and focus group discussions (FGDs).

An initial desk review of gray and published literature, policy documents, and legislation was conducted between June 2021 and October 2021. Purposive and snowballing sampling was used to identify and recruit participants for the key informant in-depth interviews. The initial list of key stakeholders at national and subnational levels to interview was derived from the lists of stakeholders involved in policy development and implementation compiled from the desk review. Subsequently, in the interviews with these purposively selected participants, they were asked about any other stakeholders. This led the researchers to several other relevant stakeholders in a snowballing approach. Seventeen national and sub-national stakeholders were interviewed in total. They included eight policymakers and program heads/officers in governmental institutions, four stakeholders from non-governmental organizations involved in adolescent sexual reproductive health, one non-governmental organization involved in mental health, four development partners funding adolescent health, and one international research and policy organization in sexual health and reproductive rights. Interviews were held virtually via ZOOM^®^ or in person at various offices with observance of all applicable COVID-19 protocols (wearing face masks, hand washing, and social distancing). The choice of in-person or online interviews was left to participants to decide, amidst the COVID-19 concerns in 2021. Nine participants opted for an online interview and eight for in-person interviews. While in-person interaction is preferred in qualitative research to enable the identification of non-verbal clues, the researchers found that for senior policymakers, there was less distraction during virtual interviews as opposed to in-person interviews.

Four FGDs were held with District Health Management Teams (DHMT) and 11 FGDs with adolescents in and out of school aged 14–19 years. To recruit adolescents in school for the focus group discussions (FGDs), all junior and senior high schools in the four selected districts were mapped and stratified into rural and urban, public and private, and boarding and day schools. Schools were purposively selected in collaboration with the district education offices to ensure representation of rural and urban, and day and boarding schools. To identify and reach out-of-school adolescents for FGDs, the researchers consulted community and opinion leaders about where to find them. The FGDs with DHMTs took place at the district health directorate offices. The DHMT comprises the District Health Director, District Director of Nursing Service, District Health Public Nurse, District Accountant, District Health Promotion Officer, District Nutritionist, District Adolescent Focal Person, and a Mental Health Officer. Representations of DHMT in the FGDs are captured in Table 1.

**Table 1:** Composition of focus group discussions with district health management teams, Greater Accra Region, Ghana, 2012–2022 (*n* = 22).

FGD with DHMTs	Present at FGD	Total number of participants
FGD District 1	District Health Director, District Accountant, District Director of Nursing Services (DDNS), District Public Health Nurse	4
FGD District 2	District Health Director, District Public Health Nurse, District Accountant, District Health Promotion Officer, District Director of Nursing Services (DDNS)	5
FGD District 3	District Health Director, District Public Health Nurse, District Accountant, District Health Promotion Officer, District Director of Nursing Services (DDNS), District Nutritionist, District Adolescent Focal Person	7
FGD District 4	District Health Director, District Public Health Nurse, District Accountant, District Health Promotion Officer, District Director of Nursing Services (DDNS), Mental Health Officer	6

FGDs with in-school adolescents were conducted in their schools and with out-of-school adolescents at venues where they worked and congregated, such as car washing bays and markets. As with in-depth interviews, all national COVID-19 protocols were observed during FGDs. This included wearing masks, washing hands, socially distancing, having the discussions in well-ventilated rooms with open windows, and limiting the number of people, including members of the research team, present at a time to 10–15 people. Participation in the study was voluntary, and participants gave their written consent before any primary data collection. Additionally, for adolescents in school, consent was also sought and granted by the School Health Programme (SHEP) Coordinator of the district, who acted as guardian for adolescents younger than 18 years in boarding school. For out-of-school adolescents younger than 18 years, consent was sought from parents and guardians.

### 2.4 Data collection tools

All interviews and FGDs were informed by semi-structured question guides. Question guide for interviews with national and sub-national stakeholders focused on exploring their knowledge, experiences, and perceptions about policies and programs for adolescent sexual and reproductive health and adolescent mental health and wellbeing. It contained questions about their interests, perceived power, influence over the design and implementation of policies and programs, the status of implementation, perceptions of effectiveness, contextual facilitators, challenges, and whether they felt there was a need for de-implementation of any of the programs.

The FGD question guide for DHMTs explored how micro-level decisions for adolescent health services were made and how they were implemented at the primary care level. It also sought information on how resources were mobilized and used for adolescent health services.

The FGD question guide for adolescents explored their understanding of sexual and reproductive health, mental health and wellbeing, challenges they faced, services available to them, perceived effectiveness of these services, and the ways that the services could be improved.

The KI interview and FGD guides were developed by the research team based on the objectives of the study and informed by reviews of literature and policy documentation, as well as an in-depth understanding of the local policy context in Ghana. Question guides for interviews and FGDs were piloted and refined after a few initial interviews/FGDs.

Basic demographic details on participants’ age, sex, and location were also collected for the adolescent FGDs (see Table 2). The interview guides were translated into Twi and Ga-Adangbe, the two most common local languages in the study areas for the interviews with out-of-school adolescents. All other interviews as well as the FGDs with adolescents in school were conducted in English, which is the official language of instruction in schools and workplaces in Ghana.

**Table 2:** Demographic characteristics of adolescent focus group participants, Greater Accra Region, Ghana 2022 (*n* = 82).

Focus group discussions with adolescents	Age range (years)	Number of participants	Level of education
FGD 1 (Girls)	16–18	8	Senior High School
FGD 2 (Boys)	17–19	8	Senior High School
FGD 3 (Girls)	16–19	8	Senior High School
FGD 4 (Boys)	16–19	9	Senior High School
FGD 5 (Girls)	14–17	6	Senior High School
FGD 6 (Boys)	16–18	6	Senior High School
FGD 7 (Girls)	16–19	8	Senior High School
FGD 8 (Boys)	17–19	8	Senior High School
FGD 9 (Boys)	14–19	5	Out of School Adolescents
FGD 10 (Boys)	18–19	8	Out of school Adolescents
FGD 11 (Girls)	14–19	8	Out of School adolescents

### 2.5 Theoretical framing and approach to data analysis

To analyze the context, we drew on Leichter’s categorization of context into situational, structural, cultural, and environmental factors (25, 26). We interpreted context as not just “external” but as permeating across the macro (i.e., systems), meso (i.e., organizational), and micro (i.e., individual and interpersonal) levels (27–30). In Leichter’s categorization of context, the environmental context refers to international factors outside Ghana, such as global agreements to which the country is a signatory, the Sustainable Development Goals (SDGs), and obligations and pressures arising thereof that affect policy and program implementation. Structural factors are the “persistent features of a nation-state and provide an enduring part of the context within which public policy is made” (25). These include systems such as the economic base, political institutions, and demographic structures. The situational context describes transient and impermanent events that nevertheless can potentially influence policy and its implementation. Examples include health security issues such as the COVID-19 pandemic, communal conflict, and natural disasters. Political culture, cultural norms, religion, traditional values and institutions, and arrangements are all part of the cultural context. We conceptualized implementation as the stage where decisions that become authoritative for society (policies) have agendas that have been set, and strategies to achieve them formulated are carried into execution.

We explored the links between context and policy implementation processes. However, it is important to mention that it is not the only context that can explain policy implementation and any gaps observed. Walt and Gilson’s policy triangle framework, which stipulates that context, actors, process, and content will all influence policy implementation and ultimately outcomes (26), was important to keep in mind as the broader conceptualization within which our analysis is nested. Our exploration of context is a deep dive into one cluster of potential explanatory factors rather than all clusters of factors. In this analysis, we focused on how and why context affects program implementation (part of the process), leading to implementation gaps. We, however, acknowledge that actors and content can also influence implementation and implementation gaps.

All KI interviews and FGDs were audio recorded and transcribed verbatim by a research assistant and were checked for quality assurance by the first author. Focus group discussions, which were conducted in the local language, were translated into English and transcribed into English. The transcripts from interviews and FGDs were coded and then analyzed with the support of qualitative analysis software, QSR NVivo version 20. To ensure intercoder reliability, the first and second authors who were part of the interviews coded the transcribed interviews, and FGDs and compared their coding using the inter-comparison coding query feature in NVivo 2020. They coded the text from the transcripts in relation to the study objectives and the conceptual framework described earlier. They used both deductive and inductive methods to create the codes. The deductive codes were created based on literature reviews and the conceptual framework of the study, while the inductive codes were created after multiple readings of the transcribed texts. All other authors were involved in the data analysis and interpretation, and they reviewed the findings from the study and agreed on themes and codes for analysis.

Validity, reliability, integrity, and trustworthiness of data and findings are important considerations of research studies. To ensure the safety and wellbeing of minors involved in our research, we obtained proper consent from parents using assent forms. By doing so, we also aimed to guarantee that the adolescent’s best interests were always at the forefront of our work. In our qualitative study, validity and trustworthiness were assured through a clear and transparent approach to the steps in the data analysis, triangulation of findings between the different methods (documents, interviews, and FGDs), and implementation of measures to minimize potential bias during the data collection and analysis. In qualitative work, potential bias in data collection, analysis, and interpretation can be introduced by the social locations of researchers who may possess advanced educational and socio-economic status than participants, in our case, adolescents who took part in the interviews and FGDs. We addressed this by maintaining critical reflexivity on the researchers’ positionality and constantly being aware of how personal attributes which accorded power could have potentially affected the research process and the resultant data. Interviewers reflected on the contents and processes of data collection, including non-verbal clues, researchers’ positionality, and the resultant biases after each interview and FGD. This was done individually as unstructured notes in field diaries and in pairs after returning from the data collection in the study sites. Furthermore, all-team weekly meetings were regularly held to monitor the work, and parts of these weekly meetings were used to reflect on the analytical themes, positionality, and resultant biases and triangulate emerging findings among the respondent groups and methods.

### 2.6 Ethical considerations

Ethical approvals were obtained from the Ghana Health Service Ethics Review Committee (GHS ERC 021/05/21) and the University of Leeds (MREC 21-010 External - AdoWA project). All primary data collection was conducted following the obtaining of informed consent. Where adolescents less than 18 years old were involved, informed consent was obtained from parents or guardians. Participation was voluntary, and participants had a choice to withdraw at any time during the data collection and were assured of anonymity and confidentiality in reporting the findings.

## 3 Findings

We first present a summary of the main objectives of the Adolescent Health and Service Policy Strategy (2016–2020) and their implementation status at the end of the 5 years covered by the policy. We then present our findings on the context and how and why it influenced policy implementation, structured by the categories in Leichter’s framework.

### 3.1 Implementation of adolescent health service policy and strategy (2016–2020)

Table 3 summarizes the eight policy objectives of the Adolescent Health and Service Policy Strategy document (2016–2020) and corresponding strategies and programs planned for implementation of each objective as well as the implementation status of each planned strategy and program. From the analysis, we categorized the implementation status as (a) fully implemented, (b) partially implemented, and (c) not implemented at all.

**Table 3:** Implementation status of Adolescent Health and Service Policy Strategy document (2016–2020) objectives.

Stated policy objectives	Strategies/programs for implementation	Implementation status
1. To improve access to information on health and health services relevant to the age-and gender-specific needs of adolescents and young people to enable them to make informed decisions	1.1 Mass Media Campaign	Implemented
	1.2 Comprehensive Sexuality Education	Not Implemented
	1.3 Adolescent Ambassadorial Challenge (School Clubs)	Partially Implemented
	1.4 Social Media Strategy (Mobile App, known as the YMK App)	Partially Implemented
	1.5 Adolescent Reproductive Health Clubs	Partially Implemented
2. To build the capacity of health service providers and support staff to enable them to have the required knowledge, skills, and a positive attitude toward the provision of effective adolescent and youth-responsive services at all levels	2.1 Capacity and Skills training	Partially Implemented
	2.2 E-learning Programme	Implemented
3. To improve access to a specified package of health services that are of high quality, gender sensitive, disability-responsive in an appropriate environment at all levels	3.1 Development of adolescent health service and policy document in Braille for the visually impaired	Implemented
	3.2 School-health based services	Partially Implemented
	3.3 Safety Net Program	Partially Implemented
	3.4 Comprehensive Abortion Care	Partially Implemented
	3.5 Girls in Iron and Folic Tablet Supplement (GIFT) Programme	Partially Implemented
	3.6 Mental Health Training	Partially Implemented
4. To develop and advocate for relevant enabling environment including protective health policies, and legislative framework to support the provision of Adolescent and Youth Responsive Health Service (AYRHS) at all service delivery and management points	4.1 Advocacy for the change in conflict between age of sexual consent and age for marriage in the constitution	Not implemented
	4.2 Enforcing laws for control of exposure, marketing, and access to unhealthy products, including Tobacco, Alcohol, beverages high in salt, sugar, and unhealthy fats	Partially implemented
5. To promote partnership and inter-sectoral collaboration among adolescent and youth groups, relevant institutions and communities in the provision and utilization of Adolescent and Youth Responsive Health Service	5.1 National Adolescent Health Committee	Partially Implemented
	5.2 The Ghana Adolescent Reproductive Health Project executed by Palladium	Implemented
6. To develop innovative strategies to address financial barriers for AYRHS to strengthen research for evidence-informed policies and interventions in AYRHS	6.1 Resource Mobilization strategies through networking and leveraging with relevant institutions	Partially Implemented
7. Strengthen research for evidence-informed policies and interventions in AYRHS	7.1 Research Advisory Team	Not Implemented
	7.2 Comprehensive Research Agenda on AYRHS	Not Implemented
	7.3 WHO Web-Based Platform for Monitoring Adolescent Services to inform AYRHS policies, programs	Partially Implemented
8. To strengthen management, leadership, and support systems for AYRHS	8.1 Review of existing leadership and governance structures	Not Implemented
	8.2 Improving supply chain systems to meet regular logistic and commodity needs at facility and service delivery points	Not implemented

Fully implemented refers to strategies and programs that were executed as originally planned. Partially implemented refers to strategies and programs that were started but were halted or not fully executed for some other reasons. Not implemented at all refers to strategies and programs that were never executed. Both partially and not implemented at all strategies and programs are considered manifestations of policy implementation gaps. A Supplementary Data file with the full policy objectives, sub-objectives, and their planned implementation strategies in the Adolescent Health and Service Policy Strategy document (2016–2020) is available.

#### 3.1.1 Fully implemented

Of the 23 planned strategies and programs for implementation of the eight objectives of the Adolescent Health Service Policy and Strategy (2016–2020), only four (17%) were fully implemented. These four were the mass media campaign under Objective 1, the e-learning program under Objective 2, the development of adolescent health service and policy document in Braille for the visually impaired under Objective 3, and the Ghana adolescent reproductive health strategy executed by Palladium under Objective 5 (Table 1).

##### 3.1.1.1 Mass media campaign under strategies and programs to achieve objective 1

To achieve objective 1 of the policy, which was to improve access to information on health and health services relevant to the age and gender-specific needs of adolescents and young people to enable them to make informed decisions, several Social and Behavioral Change Communication (SBCC) strategies were to be developed. They were expected to target specific groups such as adolescents in and out of school, adolescents with a disability, adolescents in vulnerable and underserved communities, and the general population of adolescents not in any of these categories. Only one out of the five planned strategies were fully implemented. This was the strategy of using mass media messaging to influence and change the underlying norms and attitudes that perpetuate poor health outcomes for young people. Interventions included, but were not limited to, radio and TV talk shows. They also comprised mass media interventions which were community-based and were implemented with other partner agencies to improve access to health information to adolescents to help them make informed decisions.

*…We frequently have radio discussions. Sometimes there are slots that we go in for depending on what topic is on the burner. So, if this month we want to do something on STIs, we will look for radio slots. Normally, it is with Curious minds. That is on the Ghana Broadcasting Corporation (GBC) … They have a slot at the GBC, and we have been able to ride on that platform. We also work with the Ministry of Gender, Children, and Social Protection for their “Girls, Girls” Talk Show. We normally give them resource persons because it is for Adolescent Sexual and Reproductive health and gender-based violence*”

*Interview with National Level Programme Officer/Policymaker.*

##### 3.1.1.2 E-learning platform

Objective 2 of the Adolescent Health and Service Policy Strategy document (2016–2020) was to build the capacity of health service providers and support staff to enable them to have the required knowledge, skills, and a positive attitude toward the provision of effective adolescent and youth responsive services at all levels. Out of two strategies, only one was implemented, and it was an e-learning platform created for adolescent health training, which led to wider coverage and proved to be cost-effective. It was particularly useful due to the challenges of COVID-19.

*… We were running face-to-face until Covid came then we moved online. So, we do online training, these days than face to face. So out of this, we have an e-learning programme for Ghana Health Service, open to all. We’ve been able to train more service providers ever since we went online as we can reach a lot of people.*

*Interview with National Level Programme Officer/Policymaker.*

##### 3.1.1.3 Development of adolescent health service and policy document in braille for the visually impaired

The intervention to develop an adolescent health service and policy document in Braille for the visually impaired was also fully implemented. It did not appear to be affected by contextual challenges such as resource availability or the clash with cultural context that affected some other strategies and programs.

##### 3.1.1.4 The Ghana adolescent reproductive health strategy executed by palladium

Different initiatives were aimed at promoting partnership and inter-sectoral collaboration among adolescent and youth groups, relevant institutions, and communities in the provision and utilization of adolescent and youth-responsive health services. One of these was the Ghana Adolescent Reproductive Health Project, which was funded by UK DFID, executed through Palladium, and involved moving adolescent health provision from adolescent corners to adolescent delivery points.

*“… any designated place for seeing to the health of adolescents, is now classified as a service delivery point because we want to move away from the corners to a place where you do not have a specific corner. So, it can be a desk in the entire facility, but the entire facility knows that an adolescent is coming in and there is a service provider responsible for helping the adolescent through the* var*ious stages when they come. It can also be a person moving around as an outreach point… So as of the end of 2020, we had 1,674 adolescent service delivery points.”*

*Interview with National Level Programme Officer/Policymaker.*

#### 3.1.2 Partially implemented

A majority (57%), that is, 13 of the 23 planned strategies and programs were partially implemented. These included the Adolescent Ambassadorial Challenge (School Clubs), Social Media Strategy (Mobile App, known as the You Must Know (YMK) App), and Adolescent Reproductive Health Clubs and under objective 1; health service providers and support staff capacity building under objective 2; School health-based services, Safety Net Program, Comprehensive Abortion Care, Girls in Iron and Folic Tablet Supplement (GIFT) program, and Mental Health Training under objective 3; Enforcing laws for the control of exposure, marketing and access to unhealthy products including tobacco, alcohol, beverages high in salt, sugar, and unhealthy fats under objective 4; national adolescent health committee under objective 5; and resource mobilization strategies through networking and leveraging with relevant institutions under objective 6 (see Table 1). In the next sub-sections, we describe the three most prominent strategies in this large group.

##### 3.1.2.1 Adolescent ambassadorial challenge

To reach in-school adolescents, reproductive health clubs were launched across the country to target adolescents in school with age-appropriate and gender-sensitive interventions. As part of their activities, the school clubs were challenged to develop innovative ideas to promote sexual and reproductive health among young people. Consequently, an Adolescent Ambassadorial Challenge was launched in senior high schools (SHSs) in 2019, during which some adolescents in senior high schools all over the country were assembled and pitched potential solutions to adolescent health challenges. Innovative ideas included how to fight teenage pregnancy and stop substance abuse. Winners were given a cash prize of 10,000 cedis (approximately USD 840) to implement their ideas, which provided a huge incentive to ensure the success of the program.

*So as part of the club initiative, club members every year are supposed to come up with a project to promote sexual and reproductive health and then we award. Sometimes too they get funding for their projects. So, they come and pitch. Some of them may do it before they come so that they can win funds, but others too will carry it out and come and tell us what they did for the funds to be reimbursed.*

*Interview with National Level Programme Officer/Policymaker.*

##### 3.1.2.2 Capacity and skills training

Objective 2 of the Adolescent Health and Service Policy Strategy document (2016–2020) was to build the capacity of health service providers and support staff to enable them to have the required knowledge, skills, and a positive attitude toward the provision of effective adolescent and youth responsive services at all levels. Apart from the e-learning strategy, which was fully implemented, another adopted strategy was improving the knowledge, attitude, and skills of service providers and support staff in Adolescent and Youth Responsive Health Service (AYRHS). To do this, the adolescent training manual for all health workers was revised to improve the knowledge, attitude, and skills of service providers and support staff in AYRHS. This newly designed training manual involved multiple cadres of service providers.

*…we had to revise our training manual and design a training that will target the* var*ious cadres of service providers. So, we have a system modular program. We were running face to face until Covid came then we moved online. So, we do online training, these days than face to face. So out of this, we have an e-learning programme for Ghana Health Service, open to all. We’ve been able to train more service providers ever since we went online as we can reach a lot of people.*

*Interview with National Level Programme Officer/Policymaker.*

##### 3.1.2.3 Comprehensive abortion care services

Objective 3 of the policy was to improve access to safe abortion services using the CAC approach as per the laws of Ghana (Ghana Abortion Law 1985). This law stipulates that abortion is legally permitted where the pregnancy is a result of rape, incest, or defilement, where there is a substantial risk of a physical abnormality or disease occurring in the unborn child, and where continuing with the pregnancy would risk the mental or physical health or the life of the pregnant mother (31). Although the law existed, the mental and physical grounds, as indicated in the ACT where women could seek abortion services, were not readily available in public hospitals. From 2006, the protocols and standards to regulate the provision of comprehensive abortion care were completed but had not been adopted in the public health service. These services were mainly being provided by private health institutions in partnership with other non-governmental organizations, which included the provision of abortion services to the extent permitted by the law, post-abortion care, counseling, and provision of family planning services to prevent unwanted pregnancy, and linkages to other services and information and education on the dangers of unsafe abortions. In 2021, with funding from the Buffet Foundation, CAC services were integrated as part of services in reproductive and child health (RCH) in the Ghana Health Service.

*“…previously if you walked into a primary care facility and you wanted to get an abortion, you will not get providers who are designated for that. But now we have, providers and if per the law you qualify, the service will be provided for you.”*

*Interview with National Level Programme Officer/Policymaker.*

#### 3.1.3 Not implemented at all

Six out of the 23 (approximately 26%) planned strategies and programs were not implemented at all. These were comprehensive sexuality education under objective 1, advocacy for a change in the conflict between the age of sexual consent and the age for marriage in the constitution under objective 2, establishment of a research advisory team and development of a comprehensive research agenda for AYRHS under objective 7, and review of existing leadership and governance structures and improvement of supply chain systems to meet regular logistic and commodity needs at facility and service delivery points under objective 8 (Table 1).

### 3.2 Context and implementation gaps

**Figure 1:**
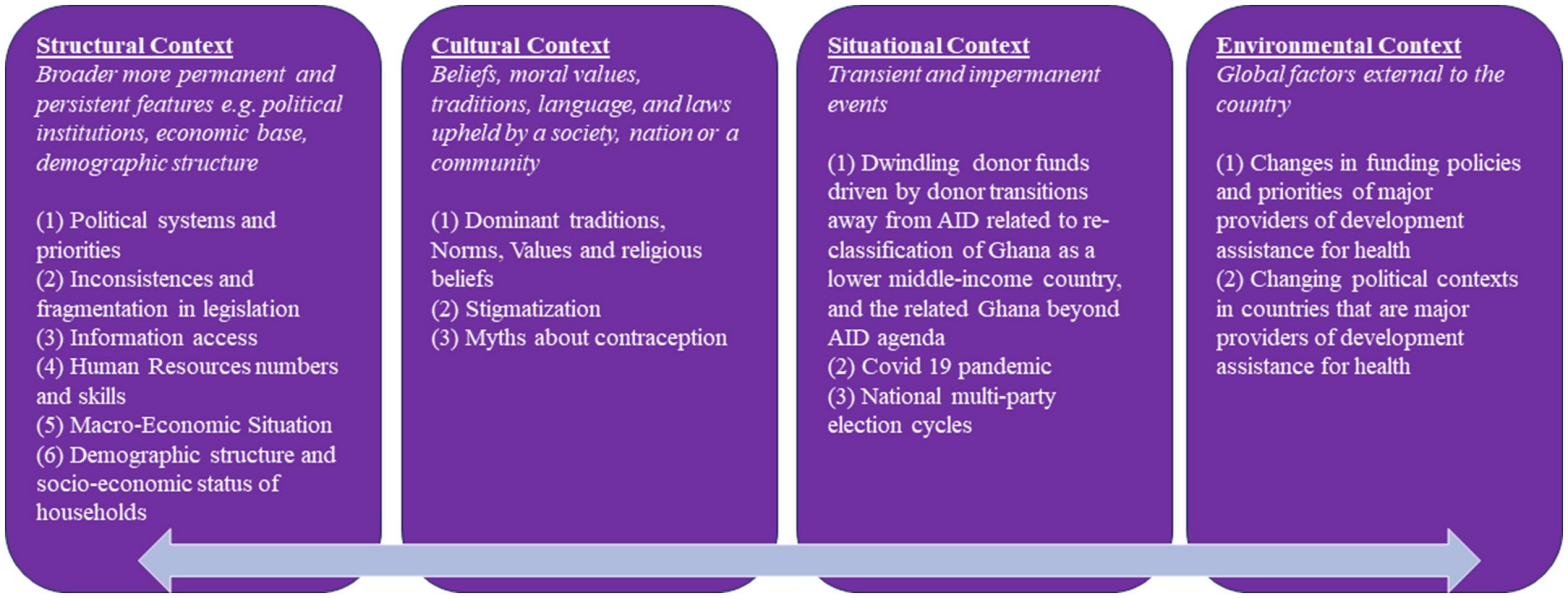
Contextual factors and adolescent health policy implementation and outcomes in Ghana.

Several contextual factors contributed to plans and strategies being partially implemented or not implemented at all. Using our conceptual framework, we present our findings on how structural, situational, cultural, and environmental factors influence adolescent health policy implementation. Our findings are summarized in Figure 1 and explained with illustrative quotes from our interviews in the text below.

#### 3.2.1 Structural context

Key structural constraints to policy implementation comprised inconsistencies and ambiguities in legislation, lack of awareness of programs and interventions, lack of human resources and skills, and a poor funding environment. In some cases, context was itself a driver of the problem that the strategy sought to address as well as a constraint to the implementation of the strategy to address the problem. Specifically, objective 4, to develop and advocate for a relevant enabling environment including protective health policies and legislative framework to support the provision of Adolescent and Youth Responsive Health Service (AYRHS) at all service delivery and management points, was effectively an effort to change constraining structural, contextual factors.

##### 3.2.1.1 Political system and priorities

Ghana returned to a multi-party democratic system in 1992 after over a decade of military rule. Two political parties, the National Democratic Congress (NDC) and the New Patriotic Party (NPP), have dominated democratic elections since 1992. Although the NDC labels its ideology as social democratic and the NPP as liberal democratic, their social policy priorities in health and education have been similar. Thus, the NPP initiated pro-poor socialist policies and programs such as the National Health Insurance Scheme (NHIS) (32), the Livelihoods Empowerment Against Poverty (LEAP), a cash transfer program for the bottom 20% of Ghana’s poor, and the School Feeding Programme between 2000 and 2008 (33). The NDC continued to maintain these social welfare programs after winning subsequent elections in 2008 and 2012^10^ and increased LEAP cash transfers. After winning the elections in 2016, the NPP instituted the free Senior High School (SHS) policy in 2017 (34). Adolescents are potential beneficiaries of all these social programs. Current political system and priorities could, therefore, be described as enablers rather than constraints to adolescent health policy implementation.

The constraints are driven by other structural, contextual factors that modify the effectiveness of these social safety net programs that should benefit adolescents (32). For example, even though adolescents under 18 years are to be exempted from user fees, under the NHIS, this was not always the case in practice because of inadequate funding (35). The huge unpaid bills of providers by the scheme meant that they were not eager to service NHIS clients or stock adequate quantities of medicines covered by the NHIS. Adolescents in school could sometimes not access medications with the NHIS cards at school sickbays, and adolescents out of school complained that the NHIS was not working for them and they were paying bills out of pocket.

##### 3.2.1.2 Inconsistencies and fragmentation in policies and legislation

Several acts of Parliament and legislative instruments provide frameworks, support, and guidance for adolescent health policy and service delivery in Ghana. Unfortunately, they are fragmented with resultant inconsistencies, which can sometimes lead to implementation challenges. For example, the age of consent to sex in the Criminal Offences Act 1960 (Act 29) is 16 years. The legal age at which one becomes an adult in the Children’s Act 1998 (Act 560) is 18 years (36). Policymakers in the interviews argued that this inconsistency between the age of consent and the age when a child becomes an adult and can legally marry constrained the implementation of several programs targeted at reducing teenage pregnancy and child marriages.

*“… the age of consent is 16 and then we are saying a child becomes an adult at 18. We are saying we want to prevent teenage pregnancy, but if at 16 years a teenager can give consent to have sex without any legal implication, then you realize that it’s going to be a challenge.”*

*Interview with Development Partner.*

The interviewees wanted the age of consent to be raised to 18 years to be consistent with that of the age at which one becomes an adult to provide clarity and effective support to programs that seek to prevent teenage pregnancies and child marriages.

Another example of inconsistent and or conflicting legislation at the time of the study was Ghana’s Criminal Offences Act (1970). Section 57:1 of the Ghana Criminal Offences Act of 1970 states that “a person who attempts to commit suicide commits a misdemeanor” (37). This law implies that anyone who attempts to commit suicide and fails is liable to arrest and prosecution and could face criminal sanctions upon conviction. This was at variance with the Mental Health Act 846 of 2012, which seeks to ensure the rights of persons with mental disorder and suicidal behavior. Stakeholders in mental health have been advocating for the decriminalization of suicide but have not had any success in repealing the law in the Ghana Criminal Offences Act of 1970 at the start of our data collection. The use of words such as “idiots” and “imbeciles” in the legal language of the law to describe people living with mental health conditions also worsens the stigma associated with persons with mental health challenges.

“… *if you attempt suicide and you fail, woe betides you! You are going to jail. That is certainly a policy that is not good enough. We are making the effort to repeal it in conjunction with some NGOs.”*

*Interview with National Level Programme Officer/Policymaker.*

To change acts of Parliament often takes long-term engagement, effort, and skills in policy advocacy and intersectoral effort that goes well beyond the health sector. The failure to resolve some of these inconsistencies, such as changing the conflict between the age of sexual consent and age for marriage in the constitution in the medium-term frame (5 years) of the implementation of the adolescent health policy, was therefore perhaps not surprising. Similarly, enforcing laws for the control of exposure, marketing, and access to unhealthy products, including tobacco, alcohol, and beverages high in salt, sugar, and unhealthy fats, were only partially implemented.

On a positive note and confirming that long rather than short-term effort is needed to address some contextual challenges, such as the need to change legislation, on 29 March 2023, the Parliament of Ghana approved amendments to the Criminal Offences Act of 170, which previously made attempted suicide a criminal offense (38). Individuals who attempt to take their life will now be regarded as needing mental health support (38). The Suicide Law is currently waiting for assent from the President to be repealed. The successful change in this law followed sustained advocacy from civil society organizations as well as the engagement of key members of the Parliament who were initially opposed to the repealing of the Law.

##### 3.2.1.3 Information access

While several policies on adolescent health exist, there was a general lack of information provision about these policies on adolescents. In all the FGDs with adolescents, none of them could identify any policy in place for adolescent health and development. At the primary care level, some health providers were not aware of the various policy and implementation strategy documents for adolescents. The You Must Know (YMK) app was developed by the Ghana Health Service, with support from the UK Foreign, Commonwealth, and Development Office (FCDO) and the United Nations Fund for Population Activities (UNFPA), to enable adolescents to have easy access to information on Adolescent Sexual and reproductive health (ASRH) as well as adolescent mental health (AMH) and wellbeing on their mobile phones, had very low usage. Only approximately 1,200 downloads had been recorded at the time of the study in 2021. Although this app has trained adolescent health workers to provide counseling through a live chat feature, the main targets of the intervention, i.e., adolescents of 15–19 years, could not access this application while in school. Junior and senior high schools in Ghana, whether day or boarding, generally do not allow pupils to bring and use personal phones in school. Moreover, even at home, not all adolescents in Ghana have access to their own mobile phones.

*“…The challenge is that those in school cannot use it because GES does not allow mobile phones. So that is the challenge but those who have finished school and all that, they have access. So it is through demand creation… we are creating a lot of demand for it and we are getting funding from USAID to create the demand for it*.”

*Interview with National Level Programme Officer/Policymaker.*

##### 3.2.1.4 Human resource numbers and skills for program implementation

In the 2016–2020 Adolescent Strategy document, the strategy for adolescent mental health was the “integration of mental health into all adolescent health services.” The provision of basic mental health services, including suicide prevention and care, was expected to be done at all adolescent service points.

*“… so every service provider who sees to adolescent is supposed to do this basic mental status exam, teach coping strategies and mechanism, teach life skills, teach some social support for those with drug abuse, sexual based violence victims, those with suicidal tendencies, those who are depressed, and other mental health conditions and they have to manage drug abuse, conditions related to stress, emotional behavior, and all those things…”*

*Interview with National Level Programme Officer/Policymaker.*

Interviews with frontline health workers at primary health facilities showed that only a few facilities offered these services because of inadequate numbers of community mental health officers trained to assess, diagnose, and provide primary mental healthcare. The few available were inequitably distributed, with urban primary care facilities better resourced than rural ones. Psychiatric nurses, similarly, were only available in a few sub-district health centers in all four study districts. Other mental health professionals, such as psychiatrists, psychologists, and occupational therapists, were not available at the primary care level, and very few at referral level facilities.

*“…at every district, we have a psychiatric nurse, so that’s not bad. The other challenge we have is with psychologists and clinical psychologists. The numbers are better now than some ten years ago but we need more. This country requires not less than 100 clinical psychologists and as we speak, in the public sector it’s just probably just about 20. We should have not less than 200 psychiatrists. The number now is around 50. The numbers are woefully inadequate. And I really cannot envisage a time when we have adequate numbers of psychiatrists, so we require the middle-level cadre.”*

*Interview with National Level Programme Officer/Policymaker.*

For the same reasons of inadequate human resource capacity, a research advisory team to develop a comprehensive research agenda was not implemented at all.

*“…Research is one of our weakest areas. Aside from supporting students who come around to collect data, we hardly on our own do we initiate, we do not. We hardly do any research in adolescent health.”*

*Interview with National Level Programme Officer/Policymaker.*

##### 3.2.1.5 Weak macro economic base and funding for adolescent health

Ghana’s Gross Domestic Product (GDP) *per capita* in current US dollars for 2021 was estimated by the World Bank at US$ 2,363 (39). Despite the government’s commitment to the Abuja Declaration in 2001, which recommended 15% of the government’s budget to be channeled to health, the target had not been attained (40). The Government of Ghana’s budget allocation for health in 2022 was only 7.6% of the total budget. This was grossly inadequate with regard to the amounts needed to support all health programs and interventions, including adolescent health (41).

Funding for implementing adolescent health programs in the policy we studied was mainly through Development Assistance for Health (DAH). Successful initiatives such as the Innovative adolescent programming, “You Only Live Once (YOLO)” television show, and the Adolescent Ambassadorial Program, where adolescents in senior high schools across the country were assembled to pitch solutions to adolescent health challenges, required continuous funding. However, their longevity came to a halt after the policy period and the end of the external funding. YOLO only came back on TV screens in 2023 when new external funders supported it.

A national adolescent health committee was instituted but funding constraints meant that the committee was not able to meet as regularly as it was supposed to.

*“… our programs have been heavily donor-driven. Government only pays the salaries and provides the structures. The operational things, we do not have adequate funds for them. When somebody is abused, service providers are forced to charge the victims to go and testify in court. We have nice policies…we spend so much to develop the policies and we do not implement them”*

*Interview with National Level Programme Officer/Policymaker*.

At the sub-national level, District directors of health across the selected study sites also raised the same challenge of the lack of consistent long-term funding for adolescent health policies and programs raised by national-level actors. They complained about regular stockouts of essential logistics and commodities needed at service delivery points, including poorly equipped adolescent health corners.

*“Unfortunately, we have beautiful policies, but when it comes to resourcing the adolescent sexual and reproductive health area, we do not see that. There aren’t any step-down funds. So we are not able to do much for the adolescents. Take this adolescent corner for example, it is the only one in the district, but you’ll realize that there’s very little here. There is not a place for adolescents to even sit to comfortably watch a film or a documentary that will help them*.

*FGD with District Health Management Team.*

The high dependence of adolescent health policies and programs on DAH for implementation meant that providers of DAH had a lot of power to influence implementation priorities. The inadequate government funding meant that scaling up and sustained implementation of policies and programs was often not done.

*“…we have all these beautiful policies, and that’s what we are good at as a country but when it comes to implementation, there’s a gap and it’s not just a small gap, it’s a huge gap. So, until we start looking at the implementation of the policy by some quantum of funds, then we will still be where we are 10 years from now.”*

*Interview with NGO in ASRH.*

Developmental partners agreed about the inadequate government funding but reiterated that their funding is supposed to identify what works and that it remains the government’s responsibility to scale up and sustain programs.

*“… a lot of the funding comes in to address specific gaps or bottlenecks… We put funding in a situation to demonstrate that with proper funding and support, there are successes that should be replicated by the government. The issue has always been whether there is funding for replication so… at the end of the day, a lot of it is donor driven and that makes the achievement of the policy somewhat not as we would want it to be.”*

*Interview with Development Partner.*

The National Health Insurance Scheme (NHIS) is a government-funded mechanism for ensuring universal health coverage in Ghana and could potentially support some aspects of adolescent health service delivery. However, it faced challenges. Long delays in reimbursement of service providers by the government have led to some government health facilities refusing to take clients on the NHIS. In two out of the three out-of-school adolescent FGDs, adolescents indicated that services at public facilities that were supposed to be covered by the NHIS were not free. In addition, there were frequent stockouts of covered medicines, leading clients to purchase them out of pocket. Infrastructural challenges also meant that adolescent health corners were not very operational. None of the districts in the study sites that were visited had a functional adolescent health corner.

*“You know something? Nowadays, if you take your health insurance to the hospital, they do not take care of you well. It exists only in name. It does not work. You cannot take your health insurance card to the hospital. If you go with your health insurance, you will only be given “Paracetamol.” [Translated from Pidgin to English]*

*FGD with out-of-school adolescent boys*.

Adolescents in school also expressed similar concerns about not being able to access medications with their NHIS cards at school clinics run by the Ghana Health Service under the School-Based Health Services Programme. This was the reality in all schools visited during this study. While adolescent health services were available, medicines were usually out of stock, and adolescents in school had to pay out of pocket for medications.

*“…the last time, I was not feeling well. It was my stomach. So, I went to the school nurse. She gave me milk of magnesia and vitamin C and she said I will pay GHS 6. When I’m sick again, I will not go to the sickbay again because when I am coming to school, my mum does not give me money to buy medicines.*

*FGD with in-school adolescent girls.*

To strengthen research for evidence-informed policies and interventions in AYRHS, some attempts were made to generate evidence to inform policies and interventions through a WHO Web-Based Platform for Monitoring Adolescent Services. This was, however, implemented in only a few facilities. The Ghana Health Service did not have the funding to conduct robust research in adolescent health interventions.

*When you want to measure perceptions of quality, you have to do a survey. So, it happens but in pockets. During our routine monitoring, we use the WHO Web-Based Platform for Monitoring Adolescent and Youth Friendly Health Service… That gives us an idea whether the training we are doing is being translated into practice… So that we can have the perception of quality in real-time without doing a survey. But the challenge with that one is that it is only in 22 facilities.*

*Interview with National Level Programme Officer/Policymaker.*

##### 3.2.1.6 National demographic structure and socio-economic status of households

The population of Ghana, according to the 2021 census, was almost 31 million, a 5-fold increase from 1960. Adolescents account for 22% of the population, and young people below the age of 18 years account for 41.8% of the population (42). This demographic structure means that there is a high dependency ratio. This is coupled with high unemployment rates and means that many households face socio-economic challenges. Various stakeholders, including national-level policymakers, district health management teams, non-governmental organizations, and developmental partners, indicated that the household socio-economic conditions of many adolescents left them in precarious situations of great vulnerability as far as ASRH and AMH are concerned.

*“…you can empower a young lady with information but if men come around and meet her with a very hungry stomach and offers her some help in exchange for sex, she may not be able to escape that risk if they do not have the means to make a living. That is the reality.”*

*Interview with NGO in ASRH.*

#### 3.2.2 Cultural context

##### 3.2.2.1 Norms and values

In Ghana, sex is usually not discussed between children and parents. Children who ask questions about sex are likely to be labeled as ‘bad children’. Regardless of whether it is actually practiced or not, chastity is upheld as a virtue, and abstinence is expected (43). Pregnancy before marriage is deeply frowned upon. The conflicts that sometimes occurred between these deeply held values and norms and actual behavior created several challenges for both the providers and adolescents. Adolescents felt shy to access family services or express a need for contraception for fear of being stigmatized.

*Respondent 2: Sometimes the reason why we are not able to go to the hospital is because of shyness.*

*Respondent 1: You can go there to meet someone’s mother and tell her that you need a condom. It would be some way.*

*Excerpt of FGD with out-of-school adolescent boys.*

The uptake of family planning services among sexually active adolescents was hampered by various myths and misconceptions surrounding contraceptives among adolescents.

*…they say it will make them infertile and fat and if it’s an IUD, it can travel to their heart, but we know a lot of them are sexually active from our data. About 14% of adolescents are accepting family planning but we wish this could increase. So, we have been focused a lot more on addressing these myths and misconceptions.*

*Interview with National Level Programme Officer/Policymaker.*

The Girls in Iron Folate (GIFT) programme, which was initiated with funding from the World Food Programme and UNICEF to provide folic tablets each day across the country to adolescent girls in and out of school, hit several roadblocks as community members did not understand what the pills were for. Some misinformation circulated on WhatsApp that the pills were contraceptives. This led to stiff opposition from community members who asked adolescent girls to refuse the capsules. The values of chastity ingrained in Ghanaian society mean that there is usually stiff opposition to the promotion of contraceptives for adolescents because they are perceived to promote immoral behavior, and any link to that is met with contempt.

*We are having some challenges with the GIFT because people are saying the folic acid we give to the adolescents are laced with contraceptives so it’s also a challenge we are trying to address through social behavioral change.*

*Interview with National Level Programme Officer/Policymaker.*

To reduce adolescent pregnancy-related school dropout rates, the Safety Net Programme was implemented in collaboration with the Girls Education Unit of the Ghana Education Service and the Department of Social Welfare of the Ministry of Gender, Children, and Social Protection in 2017 to support adolescent girls who get pregnant while in school to enable them to continue their education. At the time of the study, the program had supported over 4,000 adolescent girls and had been scaled up nationally. The program also supports the mother and child until the child is 1 year old.

*… we designed a program called the safety net program …basically, when you are pregnant and you opt to keep the pregnancy, we follow you up through home visits and help you to acquire some basic things; then when you deliver and want to go back to school, we send your name to the girls’ education office and then they follow up and ensure that you go back to school and if you need any social support, we refer you to the social welfare.*

*Interview with National Level Programme Officer/Policymaker.*

Stigma at the personal and interpersonal levels, however, deterred adolescent girls from fully taking advantage of this program.

*“There is a policy in Ghana that allows young people who get pregnant to return to school and we know that it is not being practiced not because people do not know about it but because the school environment is sometimes not healthy enough. The young people themselves fear stigma. In some situations, they do not even have uniforms and other materials that will allow them to go back to school.”*

*Interview with NGO in ASRH.*

Religion is closely tied to values and norms and has played an important role in the implementation of adolescent health programs in Ghana. Ghana is a deeply religious country, with approximately 71.3% of the population professing to be practicing Christianity and approximately 19.9% practicing Islam (3). Various stakeholders indicated that the highly religious nature of the country did not always facilitate the delivery of mental wellbeing or adolescent reproductive health services.

*“…As Ghanaians in general or Africans, we focus too much on spirituality. Honestly, I’m a very good Christian but sometimes it’s too much. We go to church from Sunday to Monday. It’s all stress. Sometimes, the spirituality has some negative effects on us. When I was in form two, I remember there was a prophetess. She was a parent but then she used to come and pray with us and then one time, she told someone that “Oh I saw that your parents had an accident.” The person cried the whole day and could not focus. So, you see, some of these things, they do not help. They do not help at all.”*

*FGD with adolescent boys in school.*

There was strong opposition to the implementation of a Comprehensive Sexuality Education (CSE) Programme from religious groups, who felt the program was not congruent with the socio-cultural as well as religious values of the community. Ultimately, the policy had to be halted.

*“… just look at what happened to CSE in this country a couple of years ago when it came out. And I keep telling everybody it was only because the tag demonic was attached to it. And that boils down to our whole concept of religion and how we see things in this country. So once CSE became demonic and a satanic agenda, there was nobody ready to lift a finger or a hand to say that “Oh I understand what CSE is and I support it”*

*Interview with Donor/Developmental Partner.*

Various policymakers intimated that the resistance and confusion that followed the proposals for the implementation of the CSE program were due to misinformation and the inclusion of socially and culturally unacceptable materials from other contexts which had explicit content on homosexuality.

Adolescents, on the other hand, tended to indicate the importance of a comprehensive sex education program to enable them to make informed decisions.

*“I feel like there are a lot of restrictions even talking about sex in the presence of adults. I feel like most of these teenage pregnancies will not have resulted. Because there is no room for discussion to talk about these things, adolescents are not well informed about certain things so, it just brings up certain unwanted issues.”*

*FGD with adolescent boys in school.*

Not all adolescents, however, agreed with the need for a comprehensive sexuality education program that promotes the use of contraceptives. In two out of the four FGDs conducted with adolescent boys in school, four adolescent boys aligned their stance with that of the education service that abstinence was the only option that should be made available to adolescents.

*As for me, I would not support the campaign for sharing condoms or maybe contraceptives, but I would only support the campaign for total abstinence. I mean, where has morality run away to? We should be moral. That would be the better option to me.*

*FGD with adolescent boys in school.*

Adolescent boys in two out-of-school FGDs, on the other hand, agreed with the use of contraceptives. The advocacy for the use of contraceptives by out-of-school adolescent boys might be attributed to the fact that they are engaged in some work that allows them to buy such commodities and have some autonomy in making decisions that affect their sexual reproductive health, as compared with adolescents who were under the guidance of the parents and teachers.

#### 3.2.3 Situational context

Apart from the cultural and religious values, the failure of the Comprehensive Sexuality Education Programme was furthered by an imminent election in 2016 and the indication by many religious groups that they would not vote for the incumbent if the policy went through under their watch. Following the change in government in 2017, the next government has also been reluctant to reintroduce such a socially divisive program with widespread opposition. Even though some national-level policymakers and developmental partners indicated that there had been some consultations and the name of the program has been changed to Reproductive Health Education, reintroducing the program has been low on the government agenda, and it remains on the shelf.

Another example of national multi-party election cycles affecting the implementation of policy is the levy or taxation for mental health, referred to in the Mental Health Law. The Ghana Mental Health Act of 2012, Act 846, states in section 88 (3) that mental healthcare is free, and apart from financing through the National Health Insurance Scheme, the Minister of Finance shall prescribe the appropriate levy or taxation for mental healthcare funding through Parliament. A stakeholder in mental health indicated that even though introducing a mental health levy could potentially help improve funding for adolescent mental health, the tax had not started implementation. He explained that with the introduction of the COVID-19 levy in addition to the already existing levy for the National Health Insurance Scheme, citizens were already burdened with taxes. Introducing the implementation of the mental health levy would make the government unpopular at a time when a national election was imminent in 2024.

*“… government is not too keen on tax now because the populace also feels overburdened, and the government is responding by scrapping what they call nuisance taxes. So if, they are scrapping taxes, then it seems counterproductive to say that a new tax should be established. So, they have not been too keen on that. That is the, if you talk to them, both publicly and in private, that is their main challenge.*

*Interview with National Level Programme Officer/Policymaker.*

Ghana was designated as a lower-middle income country in 2011 by the World Bank (44). In 2017, the newly elected President of Ghana started a “Ghana beyond Aid” campaign, which sought to progressively wean the country from donor dependency toward self-sufficiency. Both of these situational factors have, therefore, reduced DAH flows, including adolescent health.

*“So of course, in Ghana, with the Ghana beyond aid agenda, donor funds are dwindling, as Ghana moves to middle income there are some donors that will focus on poorer countries and not middle-income status countries.”*

*Interview with NGO in ASRH.*

The advent of COVID-19 also meant that a lot of resources were diverted to COVID-19 interventions, leaving adolescent health programs underfunded. In addition, donor countries hard hit by COVID-19 had to redirect funding toward their own health funding. COVID-19 also affected the implementation of programs. The National Adolescent Health Ambassador’s challenge organized for Senior Secondary Schools by the Ghana Health Service had to be halted due to the pandemic and has not resumed subsequently.

*…recently, COVID has also impacted funding. US government policy is also a major factor in funding. Brexit, whether we like it or not has also caused some challenges for funding.*

*Interview with NGO in ASRH.*

#### 3.2.4 Environmental context

Several environmental contextual factors were affecting the implementation of the adolescent health policy. Brexit in the UK led to some funding cuts that affected UK overseas development assistance for health. The United States was also a substantial funder of various adolescent health programs. The election of Donald Trump in 2016 led to a change in US foreign policy that affected funding for adolescent health interventions and programs being rolled out in many low-and middle-income countries, including Ghana.

In addition, the focus of many funding agencies has changed. Stakeholders indicated that while adolescent health remains on the agenda for many funding agencies, changes in global health policy with attention being focused on pressing issues such as climate change have also diverted attention from adolescent health.

## 4 Discussion

In this study, we have presented an exploration and analysis of the implementation of the Ghana Adolescent Health Service Policy and Strategy (2016–2020), focusing on how and why context acted as a driver of some of the observed implementation gaps in policies and programs. Structural, cultural, situational, and environmental contexts all affect policy implementation gaps to varying degrees. Our analysis suggests that different contextual factors often acted in an interrelated rather than independent manner. For example, structural challenges related to resource constraints in the macro-economic context of a lower middle-income country were exacerbated by the situational, contextual challenge of COVID-19. The COVID-19 response influenced the diversion of already inadequate funding from adolescent health and other programs and sectors, due to the urgent need to deal with the pandemic (45). The situational context was compounded by environmental contextual factors affecting bilateral DAH policies, such as Brexit in the UK and the election of Donald Trump in the US.

Explicit cultural norms that promote chastity and abstinence among adolescents meant that programs such as the Comprehensive Sexuality Programme were frowned upon and were unable to move into implementation, despite the reality of some adolescents being sexually active. Norms and values also sometimes created barriers for adolescents who wanted to access adolescent health services because of the stigma associated with sexual activity in adolescents (18, 46). Our findings are similar to other studies that have examined the multiple levels of influence on adolescents’ sexual and reproductive health decision-making (18, 47). These studies found that the communities had mostly negative perceptions of adolescent sexual activity. Most women and young girls felt that they were expected to behave modestly to gain respect from the community. At the same time, they were inadequately informed about sexual and reproductive health. These attitudes surrounding sexual and reproductive health have impacted policymaking and implementation for adolescent health. In the study by Challa and colleagues, sexual health education was identified as being necessary. However, personal opinions of policy actors about adolescent sexual health impacted the implementation of sexual and reproductive health policies that could address some of these needs (47).

Our finding that context is an important determinant of gaps in policy implementation confirms observations from other studies in Ghana (8), Uganda, South Africa, Zambia, India, Nigeria, and Honduras (19, 25, 32, 33). This effect is observed not only in adolescent health but in other health policies and programs. For example, a study that comprehensively analyzed the decisions and actions surrounding the pilot of the capitation policy for provider payment for primary care in Ghana also pointed to several contextual factors, such as technical considerations, contestations, and political responsiveness among the challenges leading to observed implementation gaps (48).

Another important observation from our study is that Adolescent Mental Health (AMH) is a neglected priority. In this study, despite the challenges, some funding for adolescent sexual reproductive health programs was reportedly trickling in from funders, and resources and funding for mental health in general and adolescent mental health, in particular, was practically non-existent. There were very limited or no mental health primary care services for adolescents in Ghana, including no specialists in mental health in primary healthcare clinics (49). In 2007, to improve access to mental health services, the Kintampo Project to train health workers in mental health was started to train community-based mental health workers. These included Community Mental Health Officers and Community Psychiatric Nurses (50). The numbers were, however, still inadequate at the time of the study as the program has stalled. In addition, because of political expediency, the mental health levy to increase funding for mental health service delivery had stalled. The lack of attention to adolescent mental health appears to be similar across sub-Saharan Africa despite their importance in the burden of disease data (11, 51, 52). A study of adolescent mental services, policies, and legislation in Ghana, Uganda, South Africa, and Zambia found that legislation, policies, programs, services, and human resources are scarce (53, 54).

One somewhat unexpected finding from our study is that contextual challenges can trigger innovation through the attempts to deal with them. For example, the advent of COVID-19 meant that capacity-strengthening sessions had to be moved online. This enabled the Ghana Health Service (GHS) to effectively incorporate adolescent health training for all cadres of health staff. The internet allowed the training of a larger number of health workers with comparatively fewer resources than required for in-person training. Resource-constrained countries can leverage the utility of internet-based online training to strengthen the capacity of healthcare professionals to offer adolescent-friendly services. In this regard, social media can also be leveraged to create awareness and drive attention to policies and program interventions among healthcare workers, adolescents themselves, as well as the general public (55).

Policy elites with influence can generate support for changes in the legislature, as the successful amendments to the Criminal Offences Act of 170, which previously made attempted suicide a criminal offense, have shown (38). Sustained advocacy from civil society organizations as well as key governmental institutions while looking out for windows of opportunity can help to align inconsistent and contradictory laws. We found that advocacy helped to bring about the revision of the old legislation in Ghana so that individuals who attempt taking their life could be regarded and treated as needing mental health support rather than as criminals (38).

Stakeholder meetings that engage key community members are also important in ensuring the success of programs. Programs such as the Comprehensive Sexuality Programme (CSE) and the Girls in Iron Folate (GIFT), which encountered roadblocks, could have benefitted from prolonged stakeholder engagements before implementation. Various studies have also emphasized the importance of community engagement in the successful implementation of adolescent programs (56, 57). Engaging community stakeholders can bring to the fore values and norms that are upheld in a society and allow for a tailoring of programs around such norms and values.

### 4.1 Limitations of the study

We acknowledge two potential limitations of our study. First, our results from adolescents primarily reflect views of school pupils aged 13–18 years and those out-of-school adolescents who are more easily accessible. We had difficulty gaining access to out-of-school adolescents as they were not in an organized setting such as the school system. Vulnerable adolescent groups, such as head porters in the study district, could not be reached due to language barriers and the inability to recognize an association to broker trust between the researchers and the participants. In the case of out-of-school adolescents younger than 18 years, parental consent could also not be easily obtained. To minimize implications, two subsequent FGDs were conducted through community leads and artisanal associations. Second, we were able to visit only four districts in the Greater Accra Region, meaning that while we were able to explore the implementation of adolescent policies in greater depth, our results primarily reflect these settings and may not be fully generalisable.

## 5 Conclusion

Analysis of context and its potential enabling or hindering effects on policy implementation should be an essential part of health policy and program decision-making and implementation to help identify potential challenges related to context and address them to reduce implementation gaps. It is important to design synergistic and integrated approaches to policies and programs and their implementation that take a holistic view of adolescents rather than fragment services and interventions into components that may operate in parallel. Bridging adolescent health policy implementation gaps requires more systemic thinking and bottom-up approaches that engage closely with adolescent perspectives and pay attention to structural and cultural contexts. Governments must find creative ways to raise sustainable funding for adolescent health programs. Furthermore, there is a need to increase advocacy for better awareness and funding of policy and program development and implementation to address adolescent health and wellbeing issues.

## Data availability statement

The raw data supporting the conclusions of this article will be made available by the authors, without undue reservation.

## Ethics statement

The studies involving humans were approved by Ghana Health Service Ethics Review Committee (GHS ERC 021/05/21) University of Leeds (MREC 21-010 External - AdoWA project). The studies were conducted in accordance with the local legislation and institutional requirements. Written informed consent for participation in this study was provided by the participants’ legal guardians/next of kin.

## Author contributions

IA and TM designed the study protocol. EA, ND, and SA led the conduct of KI interviews and FGDs supervised by IA and TM. Data analysis was done by EA, ND, and PA. EA led the conceptualization and drafting of this paper. All authors contributed to the article and approved the submitted version.
