# Supplementary material for: “We have nice policies but…”: implementation gaps in the Ghana adolescent health service policy and strategy (2016–2020)
Source: Front Public Health. 2023 Dec 12;11:1198150. doi: 10.3389/fpubh.2023.1198150 (PMC10749951; doi:10.3389/fpubh.2023.1198150)
Supplement: Supplementary file 1 [file Table_1.DOCX]

# Supplementary Data

Adolescent Health Service and Policy Strategy (2016-2020) Policy Objectives, implementation plan, desired outcomes and key targets

| **Policy Objectives** | **Plan for Implementation** | **Desired Outcomes** | **Key Targets** |
| --- | --- | --- | --- |
| **Strategic Objective 1**  **Improve access to information on health and health services relevant to the gender specific needs of adolescents and young people to enable them make informed decisions.** | - Social and behavioral Change Communication (SBCC) strategies including Comprehensive School based Sexuality Education and mass media messaging to change underlying norms and attitudes that perpetuate poor health outcomes for young people. | - Adolescents and young people are able to obtain health information and services including counselling relevant to their needs, circumstances and stage of development when seeking healthcare at various levels of health service delivery. | - 90% of adolescents and young people reached with information on   health and health services   - 90% of adolescents and young people with knowledge on SRH services and rights - Adolescent girls and young people with comprehensive knowledge on HIV by 2020 from 20% to 60% |
| **Objective 1.1**  Developing comprehensive SBCC strategy to inform, educate and promote healthy lifestyles and responsible behaviours, including sexual health in line with the National Health Promotion Policy and Strategy. | - Compiling and gathering evidence to inform the strategy development, reviewing existing SBCC materials to identify gaps, designing evidenced based SBCC programmes and interventions in collaboration with relevant stakeholders. - Printing of relevant existing and new materials and making them available for implementation. - All component areas of Adolescent and Youth Responsive Health Services (AYRHS) will be addressed under this strategy as required. |  |  |
| **Objective 1.2**  Promoting SBCC targeting adolescents in school | - Age appropriate and gender sensitive SBCC interventions (per components areas) will be designed and implemented through school-based programmes in collaboration with GES. If required, advocacy for the revision of school curriculum in line with the Policy and Strategy will be undertaken. |  |  |
| **Objective 1.3**  Promoting SBCC targeting adolescents out of school | - Age appropriate and gender sensitive SBCC interventions (per component areas) will be designed and implemented through community-based channels targeting adolescents out of school. - Appropriate community-based groups such as youth advocate networks and peer educators will be in involved in design and implementation of such SBCC interventions. - Multi-channel approaches will be used including building on existing platforms for pregnant women, adolescents, and parents. - Other social media channels that will reach our core audiences such as Twitter, Facebook, Pinterest, and WhatsApp will be used and enhanced. |  |  |
| **Objective 1.4**  Promoting SBCC targeting adolescents with disability | - The scale and scope of adolescents with disability will be assessed. - Age appropriate and gender sensitive SBCC interventions (per component areas) will be designed for people with disability and implemented through relevant institutions and organized groups. - Appropriate materials and channels (voice format, braille etc.) will be utilized depending on type of disability. The interventions will be monitored, and outcomes evaluated for re-designing. |  |  |
| **Objective 1.5**  Promoting SBCC among vulnerable adolescent including the underserved. | - The scale and scope of the problem will be assessed. - Age-appropriate and gender sensitive SBCC interventions (per component areas) will be designed and implemented targeting vulnerable and underserved adolescents and implemented through relevant institutions and organized groups. |  |  |
| **Objective 1.6**  Promoting SBCC among general population in communities | - Parents and the public will be targeted with relevant SBCC messages using multiple channels (such as interactive theatre, information vans, panel discussions etc.) to empower them to support the adolescent and young people considering their gender specific experiences such menstruation. |  |  |
| **Strategic Objective 2**  **Build capacity of health service providers and support staff to enable them have the required skills and a positive attitude for the provision of adolescent ad youth gender responsive services effectively ay all health service delivery points** | - Several strategies will be adopted to ensure that health service providers and support staff have the required knowledge, skills, and positive attitude for the provision of adolescent and youth and gender responsive services delivery points. - Needs assessment will be conducted in all component intervention area to be followed by preparation of a capacity building plan and execution of the plan covering pre-service and in-service aspects. | - Health service providers and support staff have the required knowledge, skills and a positive attitude to provide adolescent and youth responsive services relevant to the age and gender-specific needs of adolescents and young people at all health service delivery points. - Adolescents have access to specified package of health services that are of high quality, gender sensitive, disability-responsive in an appropriate - environment at all levels | - From 8% in 2016 to 70% by 2020 of targeted service providers and   support staff trained in AYRHS |
| **Strategy 2.1**  Conducting needs assessment and preparation of capacity building plan for AYRHS in the health service | - Training needs assessment on the human resource capacities for the provision of a comprehensive AYRHS will be conducted and a 5-year training plan developed accordingly. |  |  |
| **Strategy 2.2**  Ensuring adequate production and equitable distribution of health workers with appropriate skills in AYRHS | - Capacity of pre-service training institutions will be assessed, and existing curricula will be reviewed to cover all aspects of the AYRHS Policy and strategy and integrated into all health pre-service programmes. - Support will be provided to institutions which will be found to be deficient in essential aspects after the assessment. Equitable distribution of staff will be promoted at all levels of service delivery. |  |  |
| **Strategy 2.3**  Improving knowledge, attitude and skills of service providers and support staff in AYRHS | - A continuous professional training package will be designed and implemented for relevant service providers and support staff which will include post-training monitoring. The needed training manuals will be developed for various training programmes on ARYHS. |  |  |
| **Strategic Objective 3**  **Improve access to specified package of health services that are of high quality, gender sensitive, disability-responsive in an appropriate environment at all levels.** | - Enhancing AYRHS through a range of channels and delivered in ways that reach marginalized, hard to reach and vulnerable adolescents through combined interventions. - Guidelines, protocols, and tools for supervised delivery will be developed to ensure compliance at all levels. - Where possible, advocacy actions will be undertaken effectively to tackle underlying factors that increase the risk of poor health outcomes including teenage pregnancy. | - Adolescents are free from unwanted/unplanned sex, pregnancy and childbearing - Adolescents have adequate height and weight for age. - Adolescents adopt healthy lifestyles and self-care skills and are empowered to avoid use/misuse of substance. - Adolescents, especially adolescent girls, should be empowered to take decisions regarding their health. | - Reduction of adolescent mortality rate - Adolescents accessing adolescent health service by 2020 to at least 60% |
| **Strategy 3.1**  Improving access to FP services among sexually active adolescents and married adolescents | - Make access to and use of effective FP services among sexually active adolescents and married adolescents. - Enhance service and make sure that commodities are available at all service delivery channels. |  | - Increase the proportion of females aged 15-19 years using modern   contraceptive methods from 6 % in 2014 to 20 % by 2020; and among those aged 20-24 years from 21% in 2014 to 40% by 2020   - Increase the proportion of sexually active unmarried females aged 15-19   years using modern contraceptive methods from 6% in 2014 to 25% by 2020; and among those aged 20-24 years from 21% in 2014 to 40% by 2020 |
| **Strategy 3.2**  Improving adolescent pregnant girls (married and unmarried) access to timely and quality ante-natal care (ANC), skilled delivery and post-natal care (PNC). | - Existing channels will be adopted to improve access to adolescent responsive ANC, skilled delivery and PN services at all levels of care. - Maternity services will be tailored to meet the needs of teenage mothers and young fathers. - Pregnancy schools will be organized for pregnant adolescents and adolescent corners will be established in health facilities and in communities or schools with private sector involvement. - Integration of adolescent health services into the home visit/outreach package of CHPS will be promoted and CHOs will be equipped with adequate information, materials and commodities to enable them perform their duties. |  | - Increase proportion of pregnant adolescent attending the   recommended number of ANC visits from 80 per cent in 2014 to at  least 90 percent by 2020   - Increase the proportion of females aged below 20 years who deliver with the assistance of a skilled provider from 72% in 2014 to at least 90 % by 2020 |
| **Strategy 3.3**  Improving adolescent access to safe abortion services using the CAC approach as per the laws of Ghana | - Comprehensive Abortion Care services will be integrated into all adolescent sexual and reproductive health services as permitted by law. |  | - Reduce induced abortion rate among 15-19 year-old females form 17% in 2007 to 10% in 2020 and among those aged 20-24 years from 25 per cent **to 15 % by** 2020 |
| **Strategy 3.4**  Reducing adolescent health-related school dropout rate. | - Enabling pregnant and parenting girls to continue their schooling to allow them acquire education and develop skills that enhance their ability to care for themselves and their families and to increase their long-term employment opportunities. - Advocate for improvement of pre-enrolment screening of   adolescents to include other common conditions (such as sickle cell disease, diabetes, epilepsy, buruli ulcer, substance use etc.) that may lead to school dropout and prompt appropriate interventions will be provided.   - Appropriate guidelines for the screening exercise will be developed in collaboration with   Ghana Education Service. |  |  |
| **Strategy 3.5**  Strengthening school health services for adolescent | - In collaboration with GES/MOE, integrated package of school health services will be provided in all schools. - Advocacy will be undertaken to promote healthy diet and physical activity in schools and menstrual hygiene management. Standards and guidelines will be provided for rendering adolescent health service in schools. - The package of services will include HPV vaccination (females 9 to 13 years), tetanus toxoid and other vaccinations among target population in schools. |  | - Incorporation of HPV vaccine into EPI policies |
| **Strategy 3.6**  Integration of Mental Health into all adolescent health  service | - Basic mental health care, including prevention of suicide, will be provided at all adolescent health service delivery points. - Early identification of mental health conditions will be promoted by training non-medical. Personnel who work with adolescents. |  | - Reduction of prevalence of depression by age and sex |
| **Strategy 3.7**  Improving the Nutritional status of adolescents | - Adolescent girls will be specifically targeted to tackle the high prevalence of anaemia among them. Adolescents and young people will be sensitized on their nutritional needs. - Optimal nutritional composition and safety of all school meals that fall under government –sponsored school feeding programs will be advocated for. - In addition to nutritional interventions mentioned under Strategy 3.5 and 3.8, intermittent Iron-Folic Acid (IFA) supplementation will be promoted among adolescents.      - Adolescent nutrition interventions will be monitored and evaluated including the compliance to IFA supplementation. |  | - Reduction of prevalence of anaemia in women ages aged 15-19 from 47.7% in 2014 To 35% by 2020 |
| **Strategy 3.8**  Integration of Non-communicable disease (NCD) prevention into all adolescent health services | - Healthy lifestyles will be promoted and early screening, diagnosis and management services will be provided among adolescents and young people. Basic equipment (such as weighing scales, height measures, sphygmomanometers, peak flow meters, glucometers, etc.) will be provided to all service delivery points. - Enforcement of tobacco and alcohol controls in schools will be advocated for. |  | - Reduce prevalence of BMI among females undernourished (15-19) from 14.4% in 2014 to 10% by 2020 - Reduce prevalence of BMI among females Overweight/Obesity (15-19) from 8.7% in 2014 to 5% by 2020 - Age-standardized prevalence of current tobacco use among persons 15 years and older [by age and sex] - Reduce the prevalence among adolescent smokers among 15-19 years from 1.1% to less than 1% and 20-24-year youth smokers from (3.1%) to less than 2% by 2020 |
| **Strategy 3.9**  Integration of specific health needs of adolescents with  disabilities into adolescent health service delivery | - Responsive comprehensive services will be provided for adolescents with disability and the services mainstreamed into adolescent health services at all levels. This is to mitigate the challenges adolescents with disabilities face in accessing health care services. |  |  |
| **Strategy 3.10**  Provision of health services for vulnerable groups and key populations including those in hard-to-reach areas | - Specific vulnerable groups, key populations and adolescents in hard-to-reach areas will be identified and targeted for provision of AYRHS.      - CSOs / NGOs will be supported to offer adolescent health outreach services to vulnerable and key population including those in hard-to-reach areas. |  |  |
| **Strategy 3.11**  Prevention and response to harmful practices such as early and forced marriages and FGM and sexual violence against  adolescents. | - Specific at-risk adolescents such as those in early and forced marriages, child labour and those with Female Genital Mutilation (FGM) will be identified and targeted for provision of AYRHS. - CSOs / NGOs will be supported to offer adolescent health outreach services to these groups of adolescents. |  |  |
| **Strategy 3.12**  Prevention and response to violence and injuries against adolescents | - Disaster risk reduction initiatives will be promoted among adolescents (example road injuries, violence, self-harm). Services to identify, refer and prevent domestic violence will be integrated in primary health or reproductive health-care programmes for adolescents. - Advocacy activities will be undertaken to promote risk reduction (e.g., from road injuries, self-harm and violence) among adolescents and appropriate health care services will be provided. |  |  |
| **Strategy 3.13**  Improving adolescent access to STIs/HIV and  Tuberculosis services, including treatment, care, and support for all people with STIs and people with HIV. | - Treatment, care and support services for STIs/HIV and TB will be provided at all Service Delivery points, as well as post exposure prophylactic (PEP) as per national guidelines without discrimination. |  |  |
| **Strategic Objective 4**  **Develop and advocate for relevant enabling environment including protective health policies and legal frameworks to support the provision of AYRHS at all service delivery and management points.** | - The necessary enabling policy and legal environment conducive for implementing AYRHS will be developed and advocated for. - Enforcement of existing policies conducive for AYRHS will be promoted through advocacy activities, such as advocating for enforcement of tobacco and alcohol controls in schools, control of exposure of adolescents in general to unhealthy products including tobacco, alcohol, illegal substances and unhealthy foods and beverages**.** At the same time new policies/LIs will be supported for enactment to address policy issues inimical for AYRHS. - Important areas that will be addressed include early and forced marriage, Female Genital Mutilation, child labor and violence against adolescents. - The response to adolescent health will be used as an indicator of equity to create an appropriate climate for policies and laws necessary for meeting adolescent health needs. | - Ensure legal environment is in place at all levels to support the provision of AYRHS at all service delivery points. |  |
| **Strategy 4.1**  Ensuring the implementation of existing policies for  AYRHS | - Existing policies supportive of AYRHS will be identified and advocated for implementation by relevant agencies. |  |  |
| **Strategy 4.2**  Ensuring the review and enactment of new policies/LIs  to address policy issues inimical for AYRHS | - Existing policies and LIs will be reviewed and where necessary advocacy activities will be undertaken for the enactment of new ones to address policy issues inimical for AYRHS. |  |  |
| **Strategy 4.3**  Ensuring the enforcement of laws for control of exposure, marketing, importation, and access to unhealthy products including tobacco, alcohol, illegal substances and unhealthy foods and beverages high in salt, sugar, and unhealthy fats |  |  |  |
| **Strategic Objective 5**  **Promote partnership and inter-sectoral collaboration among adolescents and youth groups, relevant institutions and communities in the provision and utilization of AYRHS** | - The Government of Ghana will collaborate with bilateral and multilateral organizations in the implementation of the AYRHS. - All partners will be encouraged to spell out clearly mutually agreed duties and responsibilities in relation to outlined strategies and activities, backed by appropriate Memorandum of Understanding (MOU) to enable all collaborators play their role to their fullest potential and to facilitate all activities consistent with the AYRHS within its area of jurisdiction. - Appropriate governance structures will be established at all levels to promote joint planning and transparency, with active involvement of adolescents and young people. | - Partnership and inter-sectoral collaboration are promoted and   strengthened among adolescent and youth groups, health institutions, schools and communities in the provision and utilization of AYRHS   - Adolescents' gender and age specific views, needs and interests are identified and embedded in health policies targeting them. |  |
| **Strategy 5.1**  Ensuring better planning and coordination of projects involving Regional Coordination Council/District Coordination Council  (RCC/DCC) to avoid conflicts and improve accountability | - The composition of the National adolescent technical committee will be broadened and supported to include adolescents and youth groups among   others while multi sectorial ARYHS will be established at both regional and district levels to include representatives from RCC/DCC.   - Joint monitoring and evaluation (M&E) activities will be promoted at all levels. |  |  |
| **Strategy 5.2**  Enhancing involvement of adolescents and young people in planning, implementation and monitoring AYRHS at all levels | - An Adolescent and Youth Advisory Panel will be established as a sub-committee of the National Technical Committee with representatives from in-school and out of school adolescents and young people, including those living with disability /vulnerable, at all levels. - Appropriate TOR will be developed to guide their advisory role and used for monitoring their outputs. Adolescent participation and leadership in AYRHS - Planning and programming at all levels will be promoted and supported and equal participation of adolescent boys and girls will be ensured. |  |  |
| **Strategy 5.3**  Strengthening collaboration among GHS, GES, MoGCSP, CSOs, development partners, communities and other relevant  stakeholders at all levels. | - Collaboration among GHS, GES, MoGCSP, CSOs, development partners, communities and other relevant stakeholders at all levels will be strengthened and adolescent champions (e.g. queen mothers etc.) will be identified and supported to promote adolescent health programmes at the community level. |  |  |
| **Strategic Objective 6**  **Develop innovative strategies to address financial barriers to AYRHS** | - Innovative resource mobilization strategies will be developed and   implemented to mobilize adequate, stable, and predictable financial and in-kind resources to support the ARYHS.   - Resource support may be in the form of financing, transfer of technology and expertise and leveraging strategic partnerships with major development partners. - Funds will be mobilized from domestic and international public and private financial sources. | - Financial systems are in place at all levels to support the provision of AYRHS at all service delivery points. |  |
| **Strategy 6.1**  Mobilizing resources from internal and external sources | - Costed implementation plan for AYRHS will be developed and a resource mobilization plan will be developed and implemented. - Utilization of funds will be monitored. - Public Private Partnership (PPP) arrangements will be employed as appropriate. - Inclusion of adolescents with disabilities and vulnerabilities will be advocated for in the National Health Insurance Scheme (NHIS). |  | - Increase proportion of female adolescents age 15-19 with valid National Health Insurance cards from 52.7% in 2014 to at least 70% by 2020 |
| **Strategy 6.2**  Ensuring efficient utilization and accountability of available funds | - Adolescent health will be promoted for inclusion in the national health account and other existing accounting schemes of MOH/GHS; auditing of AYRHS funding will be done annually to ensure efficient utilization and   accountability |  |  |
| **Strategic Objective 7**  **Strengthen research for evidence-informed policies and interventions in AYRHS** | - AYRHS policies and interventions will be based on evidence, gender and age analysis, hence operational research in relevant areas will be encouraged. |  |  |
| **Strategy 7.1**  Developing and implementing a comprehensive  research agenda on AYRHS | - A research advisory team on AYRHS will be constituted to develop a   comprehensive research plan in collaboration with the Health Research and Development Division of GHS and supported to implement the research plan. |  |  |
| **Strategy 7.2**  Ensuring that research findings inform AYRHS policies, interventions and programmes | - Use of research findings will be promoted. Consequently, a database of   AYRHS will be established and updated regularly, and research findings will be disseminated to stakeholders through available media/fora to facilitate their use in programming. |  |  |
| **Strategic Objective 8 Strengthen management, leadership and other**  **support systems for AYRHS.** | - Leadership and governance for AYRHS will be strengthened to attain highest levels of transparency and accountability, and effective implementation of the programme. - Timely availability and use of appropriate gender and age-disaggregated data on AYRHS will be promoted for evidence-based decision making, as well as improving supply chain systems to meet regular logistics and commodity needs at health facilities and other service delivery points (SDPs) to facilitate efficient and effective delivery of AYRHS across the country. |  |  |
| **Strategy 8.1**  Strengthening leadership and governance for AYRHS to attain highest levels of transparency and accountability. | - Existing leadership and governance structures will be reviewed and   approaches to address gaps identified will be supported. |  |  |
| **Strategy 8.2**  Ensuring timely availability and use of appropriate gender and age- disaggregated data on AYRHS for evidence-based decision  making. | - To facilitate evidenced-based decision making, timely availability and use of appropriate gender and age- disaggregated data on AYRHS will be ensured. - Current sources of data on gender and age disaggregated data and their frequency of release will be identified and DHIMS will be revised to - incorporate data from these other sources and missing key indicators. |  |  |
| **Strategy 8.3**  Improving supply chain systems to meet regular logistics and commodity needs at health facilities and other Service Delivery Points (SDPs). | - Availability of essential commodities and logistics for AYRHS is critical for - programme implementation. - Advocacy activities will be undertaken to ensure commodity security to facilitate efficient and effective delivery of AYRHS across the country. - National, Regional and District coordinators will be trained in proper supply chain management, and supply chain officers will be trained in warehousing and logistics in partnership with Stores, Supply and Drug Management Division of GHS. |  |  |

Source: Ghana Health Service 2016, Adolescent Health Policy and Strategy (2016-2020)
